# Metaproteomic assessment of gut microbial and host functional perturbations in *Helicobacter pylori*-infected patients subjected to an antimicrobial protocol

**DOI:** 10.1080/19490976.2023.2291170

**Published:** 2023-12-08

**Authors:** Marcello Abbondio, Alessandro Tanca, Laura De Diego, Rosangela Sau, Stefano Bibbò, Giovanni Mario Pes, Maria Pina Dore, Sergio Uzzau

**Affiliations:** a Department of Biomedical Sciences, University of Sassari, Sassari, Italy; b Department of Medicine, Surgery and Pharmacy, University of Sassari, Sassari, Italy

**Keywords:** Antibiotics, fecal sample, gut microbiota, human metaproteome, infection, microbial functions

## Abstract

The impact of therapeutic interventions on the human gut microbiota (GM) is a clinical issue of paramount interest given the strong interconnection between microbial dynamics and human health. Orally administered antibiotics are known to reduce GM biomass and modify GM taxonomic profile. However, the impact of antimicrobial therapies on GM functions and biochemical pathways has scarcely been studied. Here, we characterized the fecal metaproteome of 10 *Helicobacter pylori*-infected patients before (T0) and after 10 days (T1) of a successful quadruple therapy (bismuth, tetracycline, metronidazole, and rabeprazole) and 30 days after therapy cessation (T2), to investigate how GM and host functions change during the eradication and healing processes. At T1, the abundance ratio between microbial and host proteins was reversed compared with that at T0 and T2. Several pathobionts (including *Klebsiella*, *Proteus*, *Enterococcus*, *Muribaculum*, and *Enterocloster*) were increased at T1. Therapy reshaped the relative contributions of the functions required to produce acetate, propionate, and butyrate. Proteins related to the uptake and processing of complex glycans were increased. Microbial cross-feeding with sialic acid, fucose, and rhamnose was enhanced, whereas hydrogen sulfide production was reduced. Finally, microbial proteins involved in antibiotic resistance and inflammation were more abundant after therapy. Moreover, a reduction in host proteins with known roles in inflammation and *H. pylori*-mediated carcinogenesis was observed. In conclusion, our results support the use of metaproteomics to monitor drug-induced remodeling of GM and host functions, opening the way for investigating new antimicrobial therapies aimed at preserving gut environmental homeostasis.

## Introduction

Antibiotics are extensively used to treat human gastrointestinal infections. Depending on their spectrum of activity and route of administration, antibiotics can selectively eliminate bacterial pathogens or at least decrease the luminal and mucosal bacterial density. Hence, the elimination of targeted pathogens may occur at the expense of the human intestinal microbial ecosystem, leading to various potential adverse events.^1,2^

Metagenomic analyses in human cohort studies have clarified that the gut microbiota (GM) response to antibiotic therapies may vary according to the antibiotic(s) used, duration of exposure to drugs, and lifestyle differences between individuals.^3,4^ Variation in antibiotic therapy response has also been linked to the initial GM composition.^5,6^ A recent large-scale meta-analysis of shotgun metagenomic data has added further evidence to the correlation between the stability of specific bacterial taxa and the overall GM responsiveness to therapy-induced perturbations. Specifically, the authors found that the stability of bacterial species does not correlate with their initial relative abundance, but with their capability to synthesize aromatic and non-aromatic amino acids that, in turn, supports metabolic cross-feeding and promotes cooperative microbial interaction.^4^ In mice, exposure to antibiotics for over 4 weeks has been demonstrated to generate effects also on host intestinal tissues, both directly and because of antibiotic-induced microbial imbalance.^7^ However, studies on the impact of antimicrobial therapies on gut microbial and human protein functions are lacking.

Metaproteomic approaches have the unique advantage of providing detailed and taxon-specific information about the protein functions expressed by the GM and the host within the intestinal environment. In a recently developed in vitro culture model, Figeys et al. explored the effects of four antibiotics (and other drugs) on GM biomass, taxonomy, and functions through a metaproteomic approach. Their model showed drug-dependent variations in microbial enzymatic functions and pathways mostly correlated with the abundance of the corresponding taxon. Notably, some taxa with no changes in overall abundance showed variation in specific functional features.^8^ To the best of our knowledge, metaproteomic studies assessing the impact of oral antimicrobial therapy on GM composition and functions have not yet been reported in human clinical settings.

Recently, we compared the response of the GM to two different protocols for the eradication of *H. pylori*. Composition analysis via 16S rRNA gene amplicon sequencing showed that bismuth quadruple therapy for the eradication of *H. pylori* leads to a reversible increase in Proteobacteria, Enterococcaceae, and Lactobacillaceae. The treatment included the antibiotics tetracycline and metronidazole, supplemented with bismuth subcitrate potassium and rabeprazole, and was effective in all patients under study.^9^ Here, we describe the changes occurring to GM structure and functions in a subgroup of the same patient cohort after *H. pylori* eradication therapy. The application of metaproteomics to patients’ stool samples also enabled us to monitor host fecal proteome variations and their correlation with microbial community modifications.

## Results

### Experimental design and general metrics

**Figure 1.**
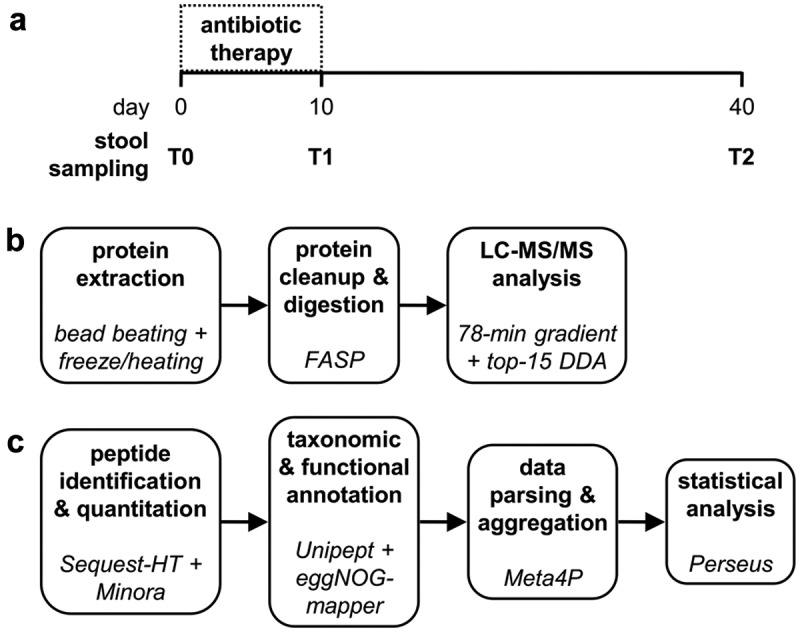
Experimental design of the study. a) sample collection: stool samples were collected from 10 patients before starting the antibiotic therapy (T0), after 10 days of therapy (T1) and 30 days after the end of therapy (T2). b) wet lab: proteins extracted from stool samples were processed according to the FASP protocol to obtain peptide mixtures, which were in turn separated by LC (78-min gradient) and analyzed with an Orbitrap Exploris 480 mass spectrometer. c) bioinformatic analysis: peptide identification and quantification from mass spectra was carried out using proteome Discoverer (with a collection of gut metagenomes and the UniProt reference human proteome as sequence databases), while Unipept and eggNOG-mapper allowed taxonomic and functional annotation, respectively. Meta4P was used to parse and aggregate data and statistical analysis (paired t test) was performed with Perseus.

**Figure 2.**
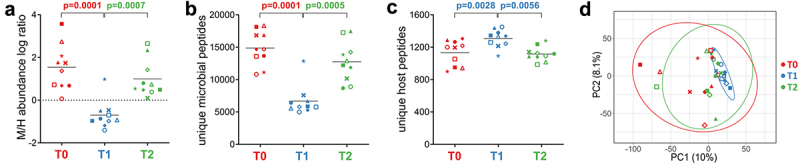
Abundance, richness, and beta diversity based on microbial and human peptide data. Each patient is marked with a different shape; each timepoint is marked with a different color. P-values obtained upon paired t-test comparison between timepoints are shown. a) scatterplot showing the log-transformed ratio between the relative abundance of microbial (M) and human (H) peptides measured in the samples. A positive M/H log ratio indicates higher abundance of microbial peptides; a negative M/H log ratio indicates higher abundance of human peptides. b) scatterplot showing the number of unique microbial peptides quantified in the samples. c) scatterplot showing the number of unique human peptides quantified in the samples. d) Principal component analysis (PCA) plot illustrating beta diversity between samples. Ellipses indicate 95% confidence level. The percentages of variation explained by the first two components are shown in x- and y-axis, respectively.

The experimental design of this study is illustrated in Figure 1. Briefly, stool samples collected from 10 patients before (T0), at the end (T1), and 30 days after the cessation (T2) of antibiotic therapy against *H. pylori* were subjected to protein extraction. Peptides obtained via filter-aided sample preparation (FASP) were analyzed by liquid chromatography-tandem mass spectrometry (LC-MS/MS) and then identified, quantified, and annotated through bioinformatic analysis. Setting the false discovery rate (FDR) threshold to 1%, a total of 53,160 peptides were quantified, of which 50,649 matched the microbial database and 2,422 matched the human database (89 were ambiguous). A taxonomic annotation was retrieved for 89% of microbial peptides (70%, 66% and 37% down to the phylum, order, and genus level, respectively), whereas 59% of them were annotated functionally. As shown in Figure 2a, the abundance ratios between microbial and human peptides were significantly different at T0 and T2 (positive values for all patients) compared to T1 (negative values in 9 out of 10 patients), suggesting a dramatically lower relative amount of microbial proteins in the stool samples collected at the end of the antimicrobial therapy. The same trend was observed when considering the unique peptide sequences identified (as an estimation of the ‘metaproteomic’ richness): a clearly higher number of microbial peptides was detected at T0 and T2 compared to T1 (Figure 2b), with an opposite result for human peptides (Figure 2c). Furthermore, the number of different microbial genera identified was significantly lower at T1 than at other time points (Supplementary Figure S1A). As illustrated in Figure 2d, principal component analysis (PCA) carried out based on all (microbial and host) peptide abundances revealed a dramatic decrease in beta diversity (i.e., diversity between individuals) at T1.

Considering the marked differences in the microbial/host abundance ratio between the time points analyzed in this study, we performed two separate differential analyses. First, we aimed to detect changes in microbial proteome expression between time points. Accordingly, we only considered microbial peptides, renormalized their abundance values (to counterbalance the global decrease in the relative abundance of the microbial component at T1), and aggregated them based on their taxonomic and functional annotations. Summed abundance values measured for taxa and taxon-specific functions in the 10 patients at T0, T1, and T2 were then compared using a paired t-test (see Methods for further details about the data analysis workflow; results of data aggregation and differential analyses are provided in Supplementary Dataset S1). In the second analysis, only the host proteins were considered, and their abundance values were normalized to obtain identical total abundances at all time points (abundance and differential data are given in Supplementary Dataset S2).

### Therapy-dependent taxonomic variations of the gut metaproteome

**Figure 3.**
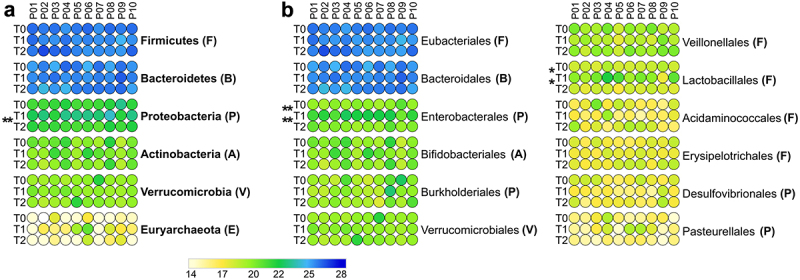
Relative abundance of top microbial phyla and orders. Peptide abundances were normalized based on the total abundance of microbial peptides in each sample, log-transformed and expressed as a color gradient (see legend in the figure bottom). The abundance of each taxon was calculated as the sum of the abundances of all peptides assigned taxonomically to that taxon by Unipept. Values measured for each of the 10 patients (P01-P10) in the three timepoints analyzed (T0, T1, T2) are shown in the heatmaps. Phylum abbreviation is given in parentheses. Statistically significant differences between timepoints are marked by asterisk(s) placed between the timepoints under comparison (* = p < .05; ** = p < .01; paired t test). a) top 6 phyla detected in the GM of the 10 patients analyzed in the study. b) top 12 orders detected in the GM of the 10 patients analyzed in the study.

**Figure 4.**
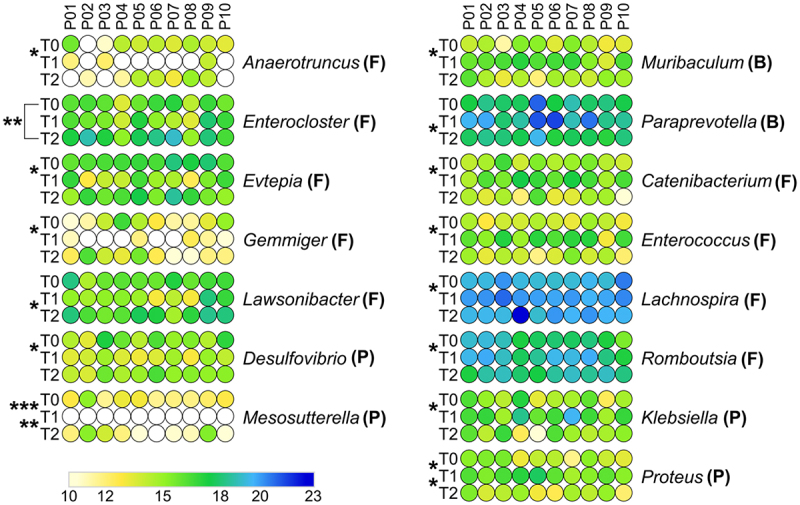
Differential microbial genera. Peptide abundances were normalized based on the total abundance of microbial peptides in each sample, log-transformed and expressed as a color gradient (see legend in the figure bottom). The relative abundance of each genus was calculated as the sum of the abundances of all peptides assigned taxonomically to that genus by Unipept. Values measured for each of the 10 patients (P01-P10) in the three timepoints analyzed (T0, T1, T2) are shown. The initial of the phylum to which each genus belongs is given in parentheses (B, Bacteroidetes; F, Firmicutes; P, Proteobacteria). Statistically significant differences between timepoints are marked by asterisk(s) placed between the timepoints under comparison (* = p < .05; ** = p < .01; *** = p < .001; paired t test). Genera with significantly lower abundance in T1 compared to T0 and/or T2 are on the left; genera with significantly higher abundance in T1 compared to T0 and/or T2 are on the right. In both cases, genera are ordered based on the phylum to which they belong and then in alphabetical order. Differential genera associated with less than 3 unique peptides are not shown.

Metaproteomic data revealed that different GM members exert different response dynamics to antimicrobial therapy, including increased, decreased, or unvaried abundance at T1 compared to T0, suggesting the existence of a variable degree of drug susceptibility and/or adaptability to therapy-dependent gut perturbations. When aggregating peptide abundance data at the phylum level, no significant changes in relative abundance were observed following the treatment (Figure 3a). However, within Proteobacteria, the order Enterobacterales was significantly increased in T1 (Figure 3b). Other members of Proteobacteria showed an opposite variation, with a decreased relative abundance in T1 (i.e., Pseudomonadales; Supplementary Dataset S1). The different levels of antimicrobial susceptibility between certain taxa belonging to Proteobacteria were also evident at the genus level (Figure 4), with a reduction in *Mesosutterella* (belonging to the order Burkholderiales) and *Desulfovibrio* (Desulfovibrionales) and an increase in *Proteus* and *Klebsiella* (Enterobacterales). Even though Firmicutes and Bacteroidetes did not show significant changes in their relative abundance after antibiotic treatment, their members exhibited a heterogeneous response to treatment. Interestingly, the abundance of Lactobacillales significantly increased after antibiotic treatment (Figure 3b). At the genus level, the relative abundances of Firmicutes members *Anaerotruncus*, *Evtepia*, and *Gemmiger* were reduced after antibiotic treatment, while *Catenibacterium, Enterococcus*, *Lachnospira*, and *Romboutsia* increased. Among Bacteroidetes, the only genus increased after drug therapy was *Muribaculum* (Figure 4).

At T2, all genera reported as decreased or increased at T1 did not show significantly different abundances compared to T0, in most cases returning to values similar to those measured at baseline (Figure 4 and PCA plot in Supplementary Figure S1B). Notably, we detected a highly significant increase in *Enterocloster* at T2 compared with T0 (Figure 4). Furthermore, Proteobacteria (among phyla, Figure 3a) and *Paraprevotella* (among genera, Figure 4) were found to decrease in relative abundance at T2 compared to T1, whereas a higher relative amount of *Lawsonibacter* was observed at T2 than at T1 (Figure 4).

### Taxon-specific protein functions variation induced by drug treatment

As reported above, the antimicrobial cocktail was more effective at limiting survival/replication and protein synthesis in certain taxonomic groups. However, in addition to direct bactericidal (bismuth and metronidazole) and bacteriostatic (tetracycline) activities, the replication rate of GM members and reshaping of their proteomes may also depend on bacterial cell adjustment to the modified metabolic fluxes across the surviving microbial community. For this purpose, the abundance of protein functions at T1 *vs* T0 was comparatively evaluated.

In total, 2,138 phylum-specific, 2,320 order-specific, and 3,214 genus-specific GM functions were detected in this study. Of these, 222, 219, and 210 functions, respectively, were significantly reduced at T1, while 52, 45, and 34 functions, respectively, were increased (Supplementary Dataset S1). At T2, all these functional features returned to levels indistinguishable from those at T0, suggesting a general recovery of the biochemical pathways and biosynthetic activities.

**Figure 5.**
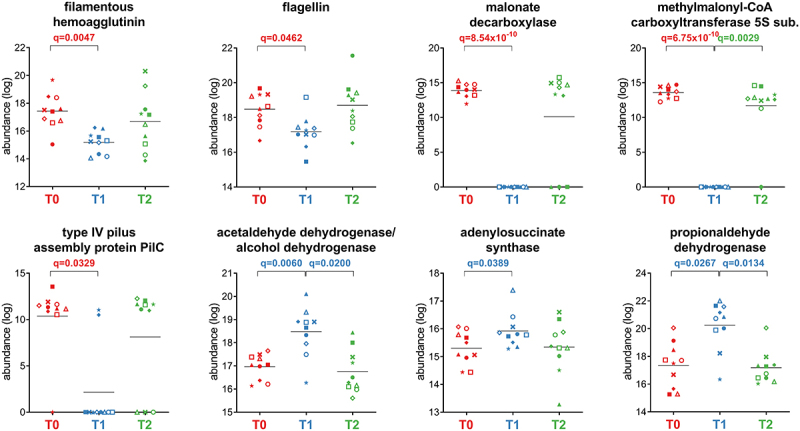
Selection of Firmicutes-specific functions with differential abundance between the timepoints analyzed. Each patient is marked with a different shape; each timepoint is marked with a different color. Q-values (FDR) obtained upon paired t-test comparison between timepoints are shown.

As shown in Figure 5, the analysis of Firmicutes functions remodeled by the treatment revealed a strong reduction in key structural proteins involved in motility and adhesion (i.e., flagellin, type IV pilus assembly protein PilC, and filamentous hemagglutinin) and in over 100 metabolic enzymes involved in amino sugar catabolism and in carbohydrate transport and metabolism (i.e., pyruvate carboxylase, 2-oxoglutarate carboxylase, glutamate dehydrogenase, acetyl-CoA C-acetyltransferase, N-acetylglucosamine-6-phosphate deacetylase, and pyruvate orthophosphate dikinase). Similarly, other anabolic and catabolic pathways were also reduced, including propionate production via the succinate pathway (methylmalonyl-CoA carboxyltransferase 5S subunit) and acetogenesis via the malonate pathway (malonate decarboxylase, malonyl-S-ACP:biotin-protein carboxyltransferase). Only 12 functions were increased at T1, with roles in purine nucleotide biosynthesis (adenylosuccinate synthase), acetogenesis via the acetaldehyde metabolism (acetaldehyde dehydrogenase/alcohol dehydrogenase and acetaldehyde dehydrogenase), and propionate production via the propanediol pathway (propionaldehyde dehydrogenase). While the overall abundance of peptides assigned to butyryl-CoA dehydrogenase (butyrogenesis) significantly increased at T1, peptides unambiguously assigned to Firmicutes, Eubacteriales, *Evtepia* and *Faecalibacterium* decreased (Supplementary Figure S3).

**Figure 6.**
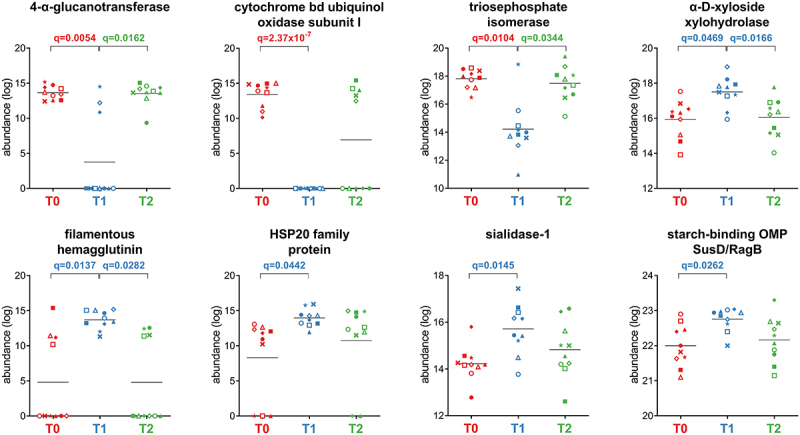
Selection of Bacteroidetes-specific functions with differential abundance between the timepoints analyzed. Each patient is marked with a different shape; each timepoint is marked with a different color. Q-values (FDR) obtained upon paired t-test comparison between timepoints are shown.

Fifty-three Bacteroidetes functions were less abundant after treatment, mostly metabolic enzymes, including cytochrome bd ubiquinol oxidase subunit I (aerobic respiratory chain), 4-alpha-glucanotransferase (glycan degradation), and triosephosphate isomerase (glycolysis), whereas only seven highly represented functions were enriched at T1 (Figure 6). The latter included three functions with specific roles in bacterial metabolism (alpha-D-xyloside xylohydrolase, starch-binding outer membrane protein SusD/RagB family, and sialidase-1), as well as stress-response mediator HSP20 family protein and filamentous hemagglutinin.

**Figure 7.**
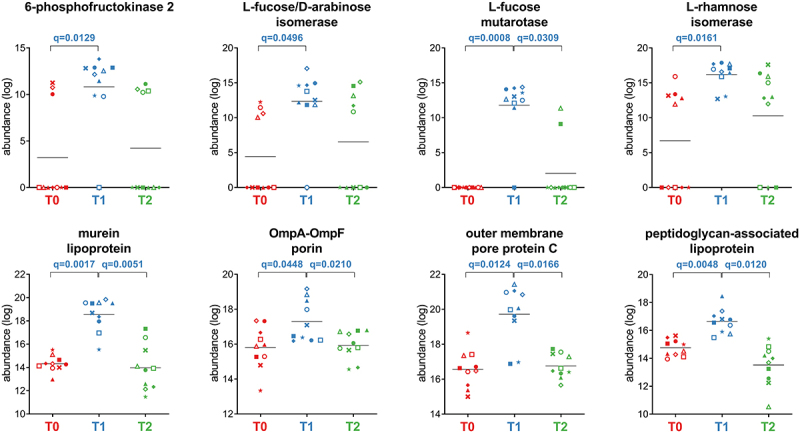
Selection of Proteobacteria-specific functions with differential abundance between the timepoints analyzed. Each patient is marked with a different shape; each timepoint is marked with a different color. Q-values (FDR) obtained upon paired t-test comparison between timepoints are shown.

Proteobacteria showed an increase in 30 functions after treatment (T1 *vs* T0), including seven outer membrane proteins (OMPs), two lipoproteins, and the metabolic enzymes L-rhamnose isomerase, L-fucose mutarotase, and L-fucose/D-arabinose isomerase (Figure 7). Twelve Proteobacteria functions were decreased at T1 (Dataset S1), including enzymes involved in amino acid metabolism (ketol-acid reductoisomerase, glutamate carboxypeptidase, alanine dehydrogenase, and glutamate dehydrogenase) and sulfur-based energy metabolism (taurine-pyruvate aminotransferase, sulfate adenylyltransferase, 3’−phosphoadenosine 5’-phosphosulfate synthase, dissimilatory sulfite reductase, also detected as significantly reduced when considering Desulfovibrionales-specific peptides).

ATP-dependent Clp protease ATP-binding subunit ClpB was the only protein assigned to Verrucomicrobia (specifically to their genus *Akkermansia*) with increased abundance at T1; further, two Actinobacteria (and *Bifidobacterium*) functions were significantly increased at T1: alpha-glucosidase and hexosaminidase (Supplementary Figure S2).

### Variation of human host proteins induced by antimicrobial therapy

**Figure 8.**
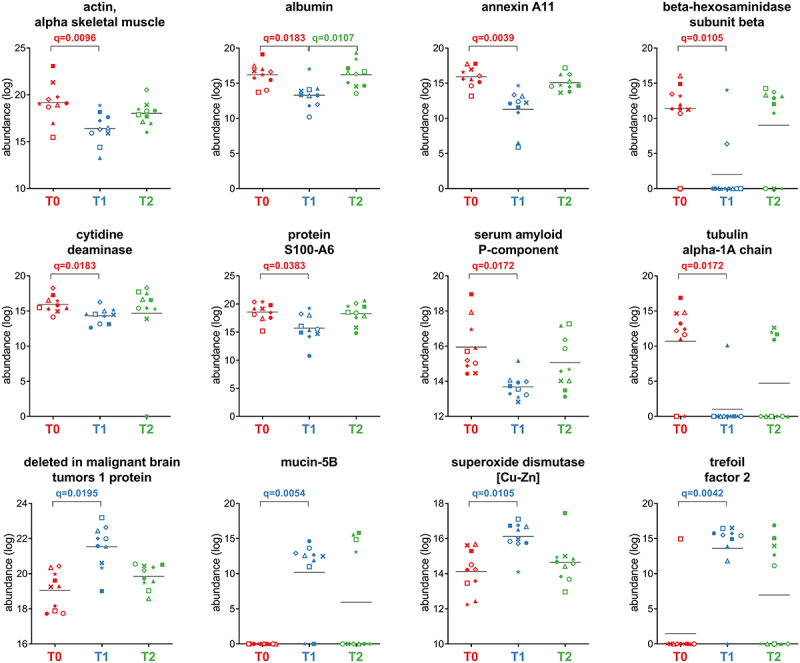
Selection of host proteins with differential abundance between the timepoints analyzed. Each patient is marked with a different shape; each timepoint is marked with a different color. Q-values (FDR) obtained upon paired t-test comparison between timepoints are shown.

After grouping the human peptides into master proteins, 572 proteins were obtained and subjected to differential analysis. Of these, 44 were found to be differentially abundant between T0 and T1. At T0, when each patient was still colonized by *H. pylori*, 22 functions were relatively more abundant than at T1 (Supplementary Dataset S2). These functions include epithelial cell structural proteins involved in actin reorganization, paracellular permeability, and mucosal barrier. The remaining 22 differential functions, including immunoglobulin polypeptides, pro-inflammatory peptidases, superoxide dismutase, trefoil factor, and DMBT-1, were more abundant at T1. A selection of the differentially abundant host proteins is provided in Figure 8.

Finally, we evaluated the correlation between host proteins and microbial genera that were found to be significantly reduced or increased at T1 compared to T0 (*n* = 17). The relative abundances of 35 host proteins that did not show significant variations between T1 and T0 were significantly correlated with the relative abundances of eight bacterial genera (Supplementary Table S1). Consistent with the differential host proteins described above, proteins involved in epithelial cell structure correlated positively with genera more abundant at T0, while six immunoglobulin polypeptides, two pro-inflammatory peptidases, meprin A, and proteoglycan 3 correlated positively with genera more abundant at T1.

## Discussion

Antibiotics are the mainstay of infectious disease treatment; however, they inherently cause perturbations in GM biomass and composition. This impact has been of steadily increasing interest because the establishment of lower diversity and/or unbalanced abundance of microbial species (dysbiosis) could contribute to further off-target effects. GM composition and functions have been linked to gastroenterological, neurologic, respiratory, metabolic, hepatic, and cardiovascular diseases.^10–13^ Hence, the GM response to antibiotics may in turn modulate its functional relations with the host physiology and health status.

The impact of antibiotic therapy on GM biomass, structure, and functions has been poorly investigated using functional meta-omics approaches (i.e., metatranscriptomics, metaproteomics, and metabolomics). In particular, metaproteomics can be used to assess the microbiome biomass and analyze community structure and its functions.^14,15^ Here, we exploited metaproteomics given its capability to quantify the bacterial taxa responsible for > 90% of the total microbiota biomass and to simultaneously investigate changes in human functions, enabling a correlation analysis of microbial concurrent variations.^15^

Exposure to the antimicrobial cocktail under study (bismuth, tetracycline, and metronidazole) led to a dramatic reduction of the GM biomass (i.e., a decrease in the number and relative abundance of peptides included in the microbial database compared to those belonging to the human database). These data are in line with those previously obtained by culture-based and metagenomic methods in antibiotic-treated mice.^16^ However, aggregating data based on taxonomic annotations showed that most of the GM bacterial members did not significantly change their relative abundances. Although we did not find significant variations at the phylum level when comparing samples collected before and after therapy, different outcomes were observed for some orders and genera. In particular, the increased abundance of the order Enterobacterales and related genera *Proteus* and *Klebsiella* at T1 versus a corresponding reduction in *Mesosutterella* and *Desulfovibrio* highlights different resistance to therapy by taxa assigned to different Proteobacteria classes: Gammaproteobacteria (Enterobacterales, *Proteus*, *Klebsiella*), Beta-proteobacteria (Burkholderiales, *Mesosutterella*), and Deltaproteobacteria (Desulfovibrionales, *Desulfovibrio*). This latter class has recently been assigned to the new phylum Desulfobacterota, which comprises an ecologically and metabolically diverse group of bacteria best known for dissimilatory sulfate reduction and previously described as susceptible to bismuth.^17,18^ Notably, the genera *Proteus* and *Klebsiella* are well recognized as human pathobionts, usually proliferating following antibiotic treatment.^19,20^

Antimicrobial therapy also led to a significant increase of *Muribaculum*, a recently described genus with specific functional features, including benzoate resistance and nitrogen utilization, which distinguish it from other Bacteroidales genera.^21^ As *Proteus* and *Klebsiella*, *Muribaculum* was as well reported to behave as pathobiont in a dysbiotic GM.^22^ Among the Firmicutes members, the increase of *Enterococcus* is of particular interest, given its pathogenicity features in antibiotic-treated patients.^23^ However, our analysis also showed an increase in other Firmicutes genera (namely, *Lachnospira* and *Romboutsia*) that are normally associated with healthy conditions. *Lachnospira* enrichment following antibiotic treatment was associated with the significantly higher abundance of stress response protein GroEL and other 2 functions: cysteine-S-conjugate beta-lyase and UDP-N-acetylmuramoyl-tripeptide – D-alanyl-D-alanine ligase. We hypothesize that the increase of the former enzyme might be related to the enhanced resistance of *Lachnospira* to the oxidative stress induced by the metallodrug bismuth.^24^ In fact, bismuth detoxification processes, in eukaryotic and prokaryotic cells, involve the induction of glutathione-S-transferase catalyzing the formation of glutathione S-conjugates.^25^ The glutathione S-conjugate is then successively converted to cysteinylglycine S-conjugate and cysteine S-conjugates that, finally, can be transformed into the corresponding thiols, pyruvate, and ammonia by cysteine-S-conjugate beta-lyases.^26,27^ Notably, also UDP-N-acetylmuramoyl-tripeptide – D-alanyl-D-alanine ligase seems to be involved in resistance of *Lachnospira* to the anti-*H. pylori* treatment, specifically against metronidazole activity. In line with the significant increase of this enzyme, a recent study based on cell imaging, RNA-seq analysis, and biological assays suggested that acquisition of resistance to metronidazole by *C. difficile* clinical strains can be partly explained by the upregulation of genes encoding enzymes involved in UDP-N-acetylmuramyl-pentapeptide synthesis and increased peptidoglycan thickness.^28^ For *Romboutsia*, that belongs to the family Peptostreptococcaceae, lower susceptibility to antimicrobial treatment can be explained by its ability to form endospore.

The potential dysbiotic effect of the antimicrobial therapy was also evident at T2, where we detected a highly significant increase in another pathobiont, the Firmicutes spore-forming *Enterocloster*, whose post-antibiotic gut recolonization has been previously reported as compromising colon cancer immunosurveillance.^29^ According to our experimental design, we did not explore time points after T2 (30 days after the end of the eradication protocol); therefore, the possibility that higher levels of *Enterocloster* are maintained even at later stages cannot be ruled out. These data confirm and extend the dynamics observed in a slightly larger cohort of patients (including those analyzed in this work) in our recent study based on 16S rRNA gene amplicon sequencing.^9^ Specifically, in both studies we consistently found an increase in Proteobacteria, Lactobacillales, and *Enterococcus* following therapy. However, compared to the metaproteomics data, 16S rRNA gene sequencing enabled us to detect a larger number of genera reduced at T1 compared to T0. This difference in sensitivity might be due to the effects of DNA amplification, the higher complexity of the molecular targets analyzed by LC-MS/MS (including host proteins), and the use of distinct annotation, quantification, and normalization strategies. In this work, the application of a metaproteomic approach enabled us to investigate taxon-specific functional reshaping by combining taxonomic and functional annotations.

Focusing on Firmicutes, our data showed significant changes in the key enzymes involved in the production of short-chain fatty acids (SCFAs) acetate, propionate, and butyrate. The abundances of acetaldehyde dehydrogenase, involved in acetogenesis,^30^ and propionaldehyde dehydrogenase, involved in propionate production via the propanediol pathway,^31^ increased at T1 and then completely reversed after therapy suspension. Strikingly, these changes appear to counterbalance the reduction observed at T1 in propionate production via the succinate pathway^31^ (methylmalonyl-CoA carboxyltransferase 5S subunit), as well as in acetate biosynthesis via two different pathways, namely malonate (malonate decarboxylase and malonyl-S-ACP:biotin-protein carboxyltransferase)^32^ and Wood-Ljungdahl (anaerobic carbon-monoxide dehydrogenase and methylenetetrahydrofolate reductase)^33^. Another function showing a significant increase at T1 is butyryl-CoA dehydrogenase, a key enzyme of the butyrate synthesis pathway.^6^ The largest contribution to this variation relies on a single predominant and highly conserved peptide (>100 times more abundant than the other peptides assigned to this function), present in over 60 UniProt entries from bacterial species belonging to different phyla (with Firmicutes and Oscillospiraceae being the most represented phylum and family, respectively). Nevertheless, 65% of peptides assigned to butyryl-CoA dehydrogenase were annotated as encoded by Firmicutes and showed an opposite trend (reduction of abundance at T1); hence, as previously described for acetate and propionate, the eradication therapy reshaped the pool of enzymes and/or pathways leading to butyrate production. Although direct measurement of metabolites was not performed in this study, our data suggest that SCFAs might be provided at high levels to the microbial communities and the host tissues, even when the GM overall viability is harshly affected by antibiotics, contributing to maintaining metabolism and immune homeostasis.^34,35^

Fifty-three Bacteroidetes functions, mostly related to metabolism, were less abundant at T1. Interestingly, the most significant reduction was observed for cytochrome bd ubiquinol oxidase subunit I, an enzyme essential for O_2_ consumption. This oxidoreductase has also been described in strict anaerobic bacteria such as *B. fragilis*, where it is triggered by nanomolar concentrations of oxygen, stimulates bacterial growth, and appears to contribute to bacterial protection against hydrogen peroxide, nitric oxide, peroxynitrite, and hydrogen sulfide.^36–38^ In our study, this enzyme was annotated at the genus level as encoded by *Alistipes*, an anaerobe emerging as a pathobiont and correlating with different human diseases.^39^ The dramatic reduction of Bacteroidetes cytochrome bd ubiquinol oxidase subunit I might be related to the increased relative abundance of facultative Enterobacterales competing for oxygen utilization and the lowered production of hydrogen sulfide by *Desulfovibrio*, thereby limiting the Bacteroidetes requirement for this enzyme. Other Bacteroidetes functions were increased following antimicrobial therapy, mostly associated with the response to stress (i.e., HSP20) and the adaptation to catabolism of specific polysaccharides. In particular, the higher abundance of SusD/RagB (starch binding outer-membrane proteins that participate in the macromolecular machines recently termed ‘utilisomes’)^40^ suggests the importance of capturing, processing and transporting complex glycans for bacterial survival in the distal gut. Another Bacteroidetes enzyme that was significantly increased at T1 was sialidase-1. This enzyme is crucial for bacterial growth on mucin and has previously been described to be enhanced by antibiotic treatment.^41,42^ The increased availability of sialic acid, in turn, might support the metabolism and outgrowth of antibiotic-associated pathogens, which do not encode sialidases on their own. Other functions increased at T1 were related to the selective pressure toward an expanded biochemical ability to degrade glycans (including host glycans): hexosaminidase (*Bifidobacterium*), alpha-glucosidase (*Bifidobacterium*/Actino-bacteria), and multiple sugar transport system substrate-binding protein (*Ruminococcus*).

The response to the antimicrobial therapy also included an increased abundance of Proteobacteria moieties involved in L-rhamnose (L-rhamnose isomerase) and L-fucose (L-fucose mutarotase and L-fucose/D-arabinose isomerase) metabolism.^43,44^ Notably, a randomized clinical trial demonstrated that healthy volunteers fed with L-rhamnose increased their serum propionate levels.^34,43^ Hence, it is tempting to speculate that the increased Firmicutes propionogenesis suggested above (propanediol pathway) might depend on the increased availability of deoxy sugars rhamnose and fucose produced by Proteobacteria.^31^ As mentioned above for sialic acid, also fucose is reported among mucin-derived sugars released by the GM, increased upon antibiotic treatment and exploited by Proteobacteria pathobionts.^42,45^ Moreover, we observed the increase of numerous OMPs encoded by Proteobacteria, including OmpA, OmpF, OmpN, OmpE, OmpC, and OmpLC. OMPs constitute a potent permeability barrier against antibiotics and other toxic molecules and are major effectors of the bacterial cell metabolic fitness and drug resistance phenotypes.^46^ OMP variations were also evident in the proteobacterial order Enterobacterales and genus *Klebsiella* (a pathobiont), but not in other Gram-negative orders such as Burkholderiales, Bacteroidales, Pasteurellales, and Desulfovibrionales. Another variation potentially leading to pathogenetic consequences is the increased abundance of Proteobacteria murein lipoprotein (MLP) and peptidoglycan-associated lipoprotein (PLP). MLP and PLP are released in complexes with LPS and circulate in experimental Gram-negative sepsis.^47^ Finally, our data showed that the contribution of Proteobacteria to sulfidogenesis was significantly reduced at T1. To this end, we showed a significantly lower abundance of enzymes (annotated as encoded by Desulfovibrionales) that are known to produce genotoxic hydrogen sulfide (H_2_S) in the human colon using inorganic (i.e., sulfate) and organic (i.e., taurine) substrates.^48^

Another outcome of the adaptation process to antibiotic pressure may be the increased abundance of *Akkermansia* (Verrucomicrobia) complex ATP-dependent Clp protease/ATP-binding subunit ClpB (ClpB). In *Lactobacillus*, the ClpL subunit of this protease has been reported as one of the few overexpressed proteins during a 12-month adaptive evolution experiment under ampicillin pressure.^49^ Given the well-known importance of bacterial Clp proteases in enabling versatile adaptation to diverse environments and efficient stress compensation,^50^ we hypothesize that they may play a role in the survival of *Akkermansia* against the stress imposed by antimicrobial drugs.

One of the striking opportunities in metaproteomics studies is the simultaneous analyses of both microbial and host proteins. The reduction of serum amyloid P-component, a protein with well-known regulatory functions in inflammation, is in line with the restoration of the gastric mucosa after *H. pylori* eradication.^51^ Beta-hexosaminidase subunit beta, a marker of degranulation triggered by *H. pylori* with a role in bacterial pathogen clearance, and protein S100-A6, both significantly reduced, were reported as markers of *H. pylori* infection and gastric cancer. Reduced abundances of protein S100-A6, myosin-2, annexin A11, actin, tubulin alpha-1A chain, and annexin A4 were also consistent with their role in cytoskeletal rearrangements and *H. pylori* disruption of the barrier function, as previously reported.^52–56^ Furthermore, among proteins with reduced relative abundance at T1, we found fecal albumin, a good indicator of a disrupted intestinal barrier, and titin, a structural protein related to *H. pylori* infection and gastric cancer.^57–59^ Finally, cytidine deaminase activity was significantly reduced at T1. The expression of this enzyme is known to be triggered by CagA-positive *H. pylori* in infected gastric tissue, promoting the accumulation of various mutations and, in turn, carcinogenesis.^60,61^ These data also suggest that the reduction of S100-A6, titin, and cytidine deaminase, which are upregulated in gastric cancer and associated with poor prognosis, might be monitored in fecal samples as markers of recovery from *H. pylori* colonization and resolution of its pathogenetic effects. However, the increase (although not significant) observed at T2 in the relative abundance of most of these proteins raises several questions, that need to be specifically addressed by future studies following patients for longer periods of time after therapy.

We also observed an equal number of host functions that were more abundant after 10 days of eradication therapy. Most of these functions are related to the gut immune response and/or wound-healing processes, including 13 immunoglobulin polypeptides, mucin 5B, superoxide dismutase, three pro-inflammatory peptidases (carboxypeptidase A1, aminopeptidase N, and chymotrypsin-like elastase family member 2A), trefoil factor, and DMBT-1. This latter is a scavenger receptor that binds to a variety of other host proteins including secretory IgA, mucin 5B, and trefoil factor 2.^62–66^

This study has several limitations, including the quite low sample size (although the clear metaproteome profile differences between T0 and T1 allowed us to find many differential features anyway), the lack of information after 30 days following the end of therapy, an unbalanced gender distribution in the patients’ cohort, and dietary information not available for the patients under study. Future studies with larger sample size, well-balanced gender distribution and longer follow up are therefore needed. In addition, including patients with unsuccessful *H. pylori* eradication would allow the evaluation of potential correlations between starting GM structure (as well as host protein levels) and eradication rate; furthermore, a detailed food diary available for each patient would open the way to investigate the GM-mediated impact of diet on *H. pylori* eradication. The intrinsic limitations of the metaproteomic approach used in this study also need to be considered, including experimental and instrumental variability, database incompleteness, the considerable share of unknown/uncharacterized proteins (40% of the microbial peptides identified in this study), and the low percentage of sequences taxonomically annotated down to genus and species level (37% and 19% in this study, respectively). Higher standardization levels in sample collection, sample preparation and LC-MS/MS methods, as well as advancements in bioinformatic resources for data annotation, are expected to further improve robustness and informativeness of metaproteomics in the years to come.

In conclusion, careful observation of GM modulation by antimicrobial therapies can provide valuable information for precision medicine and lead to improved therapeutic approaches against infectious and noninfectious diseases. Metaproteomics is a useful tool for monitoring therapy-induced remodeling of microbial functions at the level of specific taxa. Thus, this approach might be key to investigating the effect of new tailored antibiotic-based therapies aimed at better preserving GM homeostasis and boosting correct interactions with host functions.

## Materials and methods

### Patients and samples

Nineteen adult patients diagnosed with *H. pylori* infection were recruited at the Department of Internal Medicine, Gastroenterology section, University of Sassari, Italy, in the context of a larger study described elsewhere.^9^ Patients were defined as positive for *H. pylori* infection based on its detection in gastric specimens and/or a positive ^13^C-Urea Breath Test^13^C-UBT) and/or a positive stool antigen test. The exclusion criteria were as follows: pregnancy or lactation, clinically significant diseases, recent gastroenteritis or bowel preparation for colonoscopy, history of drug or alcohol abuse, and use of antibiotics or probiotics within the month preceding the enrollment. All patients received a 10-day quadruple therapy based on Pylera® (three-in-one capsule containing 140 mg bismuth potassium subcitrate, 125 mg metronidazole, and 125 mg tetracycline, qid) and supplemented with rabeprazole (20 mg, bid) and further tetracycline (250 mg, bid) and metronidazole (250 mg, bid). At the time of enrollment, patients were instructed to return all drug containers after the completion of therapy to assess adherence. Eradication was confirmed by a negative ^13^C-UBT or stool antigen test, performed 30 days after treatment completion.

Fecal samples were collected from each patient at three time points: i) at the recruitment stage, before starting antibiotic therapy (T0), ii) after 10 days of antibiotic therapy (T1), and iii) 30 days after the end of the therapy (T2). Fecal samples were kept at 4°C for a maximum of four hours after collection and stored at − 80°C until processing. Patients who were unable to provide fecal samples at all time points (*N* = 8) or for whom the result of the final *H. pylori* eradication test was not available (*N* = 1) were excluded from the study. Finally, 30 samples were analyzed from 10 patients (nine females and one male, average age 57 years, average Body Mass Index (BMI) 25.8 kg/m^2^; see Supplementary Table S2 for further details).

The study was conducted in accordance with the Declaration of Helsinki and was approved by the Institutional Review Board of the local Ethics Committee (ASL n. 1 Sassari; Prot. No. 2358/CE). All the patients signed an informed consent form.

### Protein extraction and digestion

Protein extraction from fecal samples was performed as described earlier,^67^ with minor modifications. Extraction buffer (2% SDS, 100 mM DTT, 20 mM Tris-HCl pH 8.5; 100 µl per 50 mg of stool) and a steel bead (5 mm diameter; Qiagen, Hilden, Germany) were added to each sample. Then, samples were processed as follows: incubated at 95°C for 20 min in a thermoblock (FALC, Treviglio, Italy), incubated at −80°C for 10 min, bead beaten for 10 min (30 cycles/s in a TissueLyser LT mechanical homogenizer, Qiagen), incubated at −80°C for 10 min, incubated at 95°C for 10 min, bead beaten for 10 min (30 cycles/s), and centrifuged at 14,000 × g for 10 min.

Supernatants were collected and further processed according to a modified FASP protocol.^68,69^ Accordingly, protein extracts (30 µl each) were diluted with 370 µl of UT solution (8 M urea, 100 mM Tris-HCl pH 8.5), loaded onto an Amicon Ultra-0.5 filtration device (30 kDa cutoff; Merck, Darmstadt, Germany), and centrifuged at 14,000 × g for 15 min. The following solutions were sequentially added to the samples, which were then centrifuged at 14,000 × g for 10 min: 200 μl of UT solution, 100 μl of 50 mM iodoacetamide in UT solution (followed by 20 min incubation at RT), 100 μl of UT solution, 100 μl of UT solution, and 100 μl of 50 mM ammonium bicarbonate. Finally, samples were digested overnight at 37°C with trypsin (1 μg per sample, dissolved in 50 mM ammonium bicarbonate solution). The first eluate was collected by centrifugation (14,000 × g for 15 min). Then, 100 μl of elution solution (20% acetonitrile, 0.2% formic acid) was added to each sample, and a second eluate was collected by centrifugation (14,000 × g for 15 min) and merged with the first one. The peptide mixtures were finally concentrated using Concentrator Plus (Eppendorf, Hamburg, Germany) and sent to an external laboratory for LC-MS/MS analyses.

### LC-MS/MS analyses

Offline StageTip purification and nanoLC analysis were performed as described previously.^70^ Reconstituted peptide mixtures (approximately 5 µg) were purified using SCX StageTips, eluted in 10 µl of 500 mM ammonium acetate containing 20% acetonitrile, evaporated to dryness, and resuspended in 0.2% formic acid. A 200-ng aliquot of the peptide mixture was injected for preliminary nanoLC-MS/MS analysis. The appropriate injection volume (between 1 and 8 µl) was estimated by analyzing a 1 µl aliquot of the digest using a short LC gradient and by estimating the peptide amount based on the overall peptide signal (area under the curve). LC was performed using an EasyLC 1200 instrument (Thermo Fisher Scientific, Waltham, USA). The nanoLC column was a pulled capillary of 0.075 × 160 mm (i.d. and column length, respectively), which had been packed in house with C18 silica particles (Dr. Maisch, Ammerbuch, Germany). Peptides were loaded at 500 nl/min in mobile phase A (2% acetonitrile, 0.1% formic acid) and eluted at 300 nl/min using the following gradient: from 0% B to 25% B (80% acetonitrile, 0.1% formic acid) in 60 min, from 25% B to 45% B in an additional 10 min, then to 100% B in 8 min. The column was regenerated for 10 min at 100% B and equilibrated at 0% B for 20 min before the following injection. Two blank injections were performed between samples. A shorter gradient (45 min) was used for blank injections.

Peptides were electrosprayed in positive ion mode into an Orbitrap Exploris 480 (Thermo Fisher Scientific) using 1800 V as spray voltage. Internal calibration was automatically performed at the beginning of each run (RunStart Easy IC-on). The full scan MS parameters were as follows: scan range 375–1400 m/z, resolution 60,000, RF lens 40%, AGC target 100%, and maximum injection time 50 ms. Data-dependent acquisition was performed using the following parameters: dependent scans 15 (top-15), dynamic exclusion 20 s, charge states 2–6, and intensity threshold 5e^4^. Tandem mass spectrometry scans were acquired as follows: isolation window 1.6 m/z, resolution 30,000, normalized collision energy 30%, AGC target 100%, and maximum injection time 120 ms.

### Bioinformatic analyses

Peptide identification was carried out using Proteome Discoverer™ (v.2.5; Thermo Fisher Scientific), with Sequest-HT as the search engine and Percolator for peptide validation, setting the FDR threshold to 1%. Search parameters were as follows: precursor mass range 350–5000 Da, minimum peak count 6, S/N threshold 2, enzyme trypsin (full), maximum missed cleavage sites 2, peptide length range 5–50 amino acids, precursor mass tolerance 10 ppm, fragment mass tolerance 0.02 Da, static modification cysteine carbamidomethylation, and dynamic modification methionine oxidation. Searches were conducted in parallel against two sequence databases, namely a collection of human gut metagenomes (available at https://ftp.cngb.org/pub/SciRAID/Microbiome/humanGut_9.9M/GeneCatalog/IGC.pep.gz)^71^ and the *Homo sapiens* reference proteome retrieved from UniProtKB/Swiss-Prot (release 2021_04); proteins were categorized as ‘microbial’ or ‘human’ when belonging to the first or second database, respectively.

Offline mass recalibration and label-free MS1 quantitation were performed using Spectrum Files RC and Minora Feature Detector nodes, respectively. Optimal settings for retention time and mass tolerance windows were calculated by Minora based on the mass accuracy and retention time variance distributions. A consensus feature list was defined based on the outputs of Feature Mapper and Precursor Ions Quantifier nodes. The MS1 signals of all peptides significantly matching with at least one MS2 spectrum from at least one sample were mapped across runs and quantified by calculating the integrated area of the chromatographic peak.

Unipept Desktop (v.2.0.0) was used to carry out peptide taxonomic annotation,^72^ selecting the three available options (“equate I and L”, “filter duplicate peptides” and “advanced missed cleavage handling”). Protein sequences were subjected to functional annotation using the eggNOG-mapper web application (v.2.1.9, available at http://eggnog-mapper.embl.de/),^73^ keeping default parameters and then choosing KEGG (Kyoto Encyclopedia of Genes and Genomes) orthology (KO) information as the main functional classification.^74^ Meta4P (v.1.2) was used to parse identification, quantification, and annotation data and generate aggregated abundance tables.^75^ The abundance of a taxon, a function, or a taxon-specific function was estimated by summing the peak areas associated with all peptides having that feature among their annotations. For differential analysis of host proteins, master proteins (determined by Proteome Discoverer’s protein grouping algorithm, according to the maximum parsimony principle) were considered.

### Statistical analyses and graph generation

PCA plots were generated using ClustVis, selecting the following parameters: ln(x + 1) transformation, row centering, Pareto scaling, and Nipals PCA.^76^ The Perseus computational platform (v.1.6.7.0)^77^ was used to carry out differential analysis on aggregated abundance tables, according to the following sequential steps: abundance data were subjected to binary logarithmic transformation to approximate a normal distribution (checked afterward using the Kolmogorov-Smirnov test), peptides not reaching 75% of valid values in at least one group (for each comparison) were filtered out, missing values were replaced with a constant value calculated as the binary logarithm of the lowest peptide abundance (approximated to the nearest integer) minus 1, differential peptide abundances between groups were tested with a two-tailed paired Student’s t-test, and t-test p-values were corrected for multiple testing by calculating an FDR (according to Benjamini and Hochberg)^78^ considering q = 0.05 as the significance threshold. Scatter plots were created using GraphPad Prism (v.9). Heatmaps were generated using Morpheus (https://software.broadinstitute.org/morpheus).

## Supplementary Material

Suppl Table S1.xlsx

Suppl Table S2.xlsx

Suppl Figure S2.tiff

Suppl Figure S1.tiff

Suppl Dataset S1.xlsx

Suppl Dataset S2.xlsx

Suppl Figure S3.tiff

Supplementary file legends.docx

## Data Availability

The mass spectrometry proteomics data (including peptide identification, quantification and annotation tables) have been deposited to the ProteomeXchange Consortium via the PRIDE^79^ partner repository with the dataset identifier PXD042946.
