# Supplementary figures and images for: Metaproteomic assessment of gut microbial and host functional perturbations in Helicobacter pylori-infected patients subjected to an antimicrobial protocol
Source: Gut Microbes. 2023 Dec 8;15(2):2291170. doi: 10.1080/19490976.2023.2291170 (PMC10730194; doi:10.1080/19490976.2023.2291170)

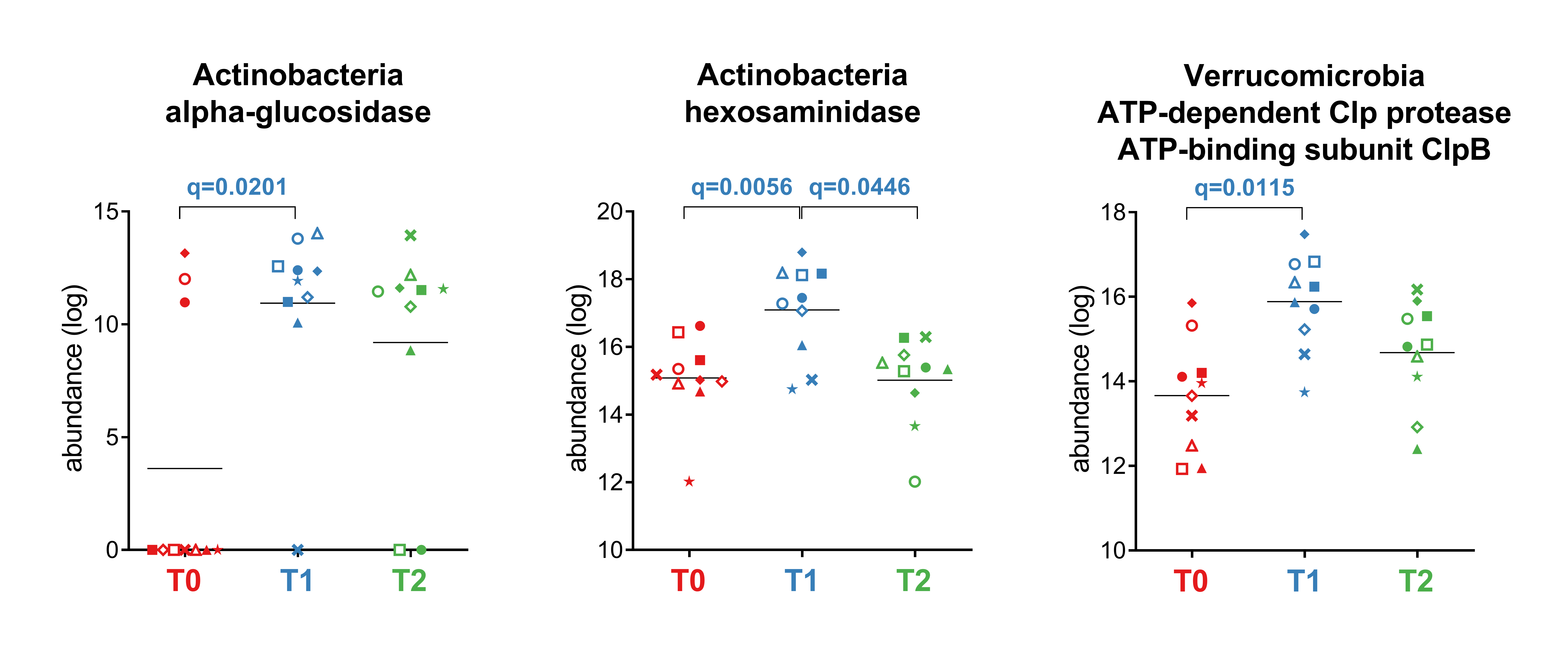

Supplement: Suppl Figure S2.tiff [file KGMI_A_2291170_SM7171.tiff]

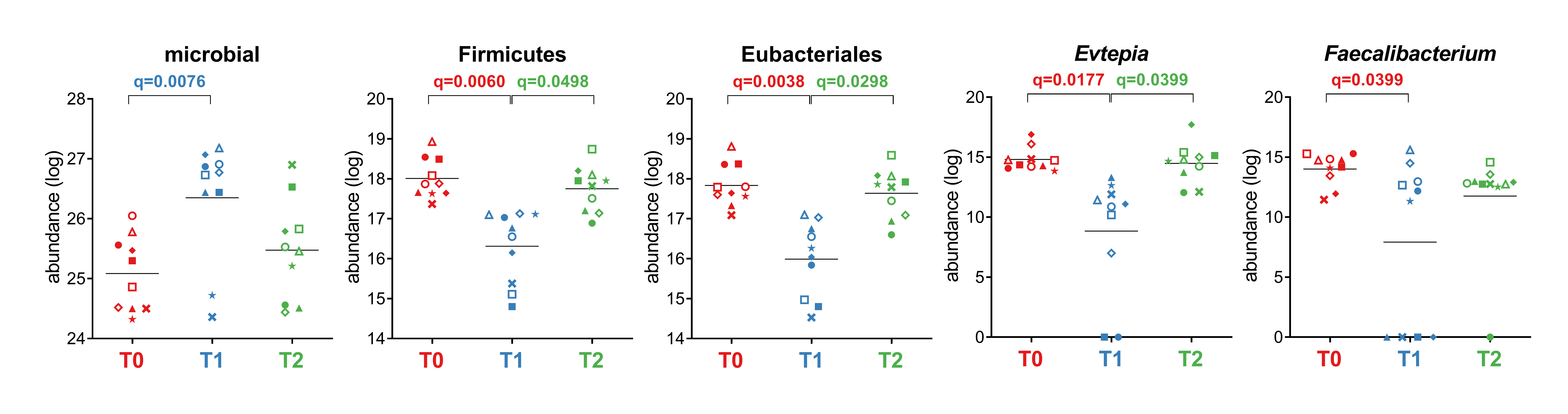

Supplement: Suppl Figure S3.tiff [file KGMI_A_2291170_SM7167.tiff]
