# Supplementary material for: Metaproteomic assessment of gut microbial and host functional perturbations in Helicobacter pylori-infected patients subjected to an antimicrobial protocol
Source: Gut Microbes. 2023 Dec 8;15(2):2291170. doi: 10.1080/19490976.2023.2291170 (PMC10730194; doi:10.1080/19490976.2023.2291170)
Supplement: Supplementary file legends.docx [file KGMI_A_2291170_SM7166.docx]

Metaproteomic assessment of gut microbial and host functional perturbations in *Helicobacter pylori*-infected patients subjected to an antimicrobial protocol

# Supplementary file legends

**Dataset S1.** Normalized microbial peptide abundances aggregated at the phylum, order, genus, KEGG KO, phylum-specific KEGG KO, order-specific KEGG KO and genus-specific KEGG KO levels. Summed abundances measured for each sample and timepoint are shown, along with the percentage of valid values per timepoint, the total peptide counts. Outputs of differential analyses, including p- and q-values, log ratios and abundance trends, are also provided. See Methods for further details.

**Dataset S2.** List of host master proteins, along with normalized abundances measured for each sample and timepoint, percentage of valid values per timepoint and total peptide counts. Outputs of differential analyses, including p- and q-values, log ratios and abundance trends, are also provided. See Methods for further details.

**Figure S1**. Diversity of microbial genera. A) Scatterplot showing the number of microbial genera detected in the samples. Each patient is marked with a different shape; each timepoint is marked with a different color. P-values obtained upon paired t test comparison between timepoints are shown. B) Principal component analysis (PCA) plot illustrating beta diversity between samples. Each patient is marked with a different shape; each timepoint is marked with a different color. Ellipses indicate 95% confidence level. The percentage of variation explained by the first two components are shown in x- and y-axis, respectively.

**Figure S2.** Selection of Actinobacteria- and Verrucomicrobia-specific functions with differential abundance between the timepoints analyzed. Each patient is marked with a different shape; each timepoint is marked with a different color. Q-values (FDR) obtained upon paired t test comparison between timepoints are shown.

**Figure S3.** Scatter plots showing relative abundance of butyryl-CoA dehydrogenase, according to different levels of taxonomic specificity. Each patient is marked with a different shape; each timepoint is marked with a different color. Q-values (FDR) obtained upon paired t test comparison between timepoints are shown.

**Table S1.** Spearman's correlation coefficients (rho) and q-values calculated by correlating the relative abundances of a selection of microbial genera (previously found as differentially abundant between T0 and T1) with those of host master proteins.

**Table S2.** Gender, age, and BMI information of the 10 patients analyzed in this study.
